# The Addition of Viriditec^TM^ Aqueous Ozone to Peracetic Acid as an Antimicrobial Spray Increases Air Quality While Maintaining *Salmonella* Typhimurium, Non-pathogenic *Escherichia coli*, and *Campylobacter jejuni* Reduction on Whole Carcasses

**DOI:** 10.3389/fmicb.2018.03180

**Published:** 2019-01-08

**Authors:** Dana Kristen Dittoe, Kristina Marie Feye, Bob Peyton, Drew Worlie, Michael J. Draper, Steven C. Ricke

**Affiliations:** ^1^Department of Food Science, University of Arkansas, Fayetteville, AR, United States; ^2^Center for Food Safety, University of Arkansas, Fayetteville, AR, United States; ^3^TetraClean Systems LLC, Omaha, NE, United States

**Keywords:** poultry, spray cabinet, aqueous ozone, peracetic acid, pathogenic reduction, ambient peracetic acid vapor

## Abstract

Currently, the most utilized antimicrobial in processing facilities is peracetic acid, PAA; however, this chemical is increasingly recognized as a hazard to human health. Preliminary evidence suggests that ozone, when introduced in a specific manner, can reduce the noxious nature of PAA. Therefore, the objective of the current study was to evaluate the efficacy of TetraClean Systems aqueous ozone, O_3_, in combination with PAA as an antimicrobial spray on whole chicken carcasses. This trial used 70 whole hen carcasses (7 treatments; 10 replications) that were inoculated in a 400 mL cocktail containing *Salmonella, Escherichia coli*, and *Campylobacter* (10^7^ CFU/mL) and allowed to adhere for 60 min at 4°C for a final concentration of 10^5^ to 10^6^ CFU/g. The experimental 5 s (4×) spray treatments included: a no treatment negative control, TW; TW + O_3_ (10 ppm), TW + PAA (50 ppm), TW + PAA (500 ppm), TW + O_3_ + PAA (50 ppm), and TW + O_3_ + PAA (500 ppm). During treatment application, ambient PAA vapor was measured with a ChemDAQ Safecide PAA vapor sensor. After treatment, carcasses were immediately rinsed in 400 mL of nBPW for 2 min. Following rinsing, the dot method was utilizing for enumeration with 10 μL of rinsate being serially diluted, plated on XLD and mCCDA agar, and incubated aerobically at 37°C for 24 h or microaerophilically at 42°C for 48 h. Log-transformed counts were analyzed using ANOVA in JMP 14.0. Means were separated using Tukey’s HSD when *P* ≤ 0.05. There was a significant treatment effect among *Salmonella, E. coli*, and *Campylobacter* counts, and a significant treatment effect among ambient PAA (*P* < 0.05). TW + O_3_ + PAA (500 ppm), reduced *Salmonella* significantly compared to TW (5.71 and 6.30 log CFU/g). Furthermore, TW + PAA (500 ppm), reduced the presence of *E. coli* significantly compared to TW or no treated control (5.57 and 6.18 log CFU/g). Also, TW + PAA (50 ppm), TW + PAA (500 ppm), and TW + O_3_ + PAA (500 ppm) significantly reduced *Campylobacter* compared to carcasses not treated (4.80, 4.81, and 4.86 log CFU/g). Lastly, the addition of ozone significantly reduced the ambient PAA when O_3_ was added to 500 ppm of PAA, as TW + O_3_ + PAA (500 ppm) produced less ambient PAA than TW + PAA (500 ppm) (0.052 and 0.565 ppm). In conclusion, the addition of ozone to PAA may demonstrated the ability to effectively reduce ambient PAA, thus increasing employee safety.

## Introduction

Currently, the United States poultry industry utilizes peroxyacetic acid, also known as peracetic acid (PAA), to decontaminate poultry within poultry processing facilities. The disinfectant, PAA, is a product of the reaction between acetic acid and hydrogen peroxide. It is a colorless acid with a strong odor. The bactericidal effect of PAA is due to it being a strong oxidizing agent of the cell membrane and other cell components (Oyarzabal, 2005). However, this chemical is corrosive and unstable. PAA is one of the most common antimicrobials used in poultry processing facilities, as it is applied in the chillers (pre-chiller, chiller, and post-chiller), part dips, spray cabinets, in and out bird washes at concentrations typically ranging from 200 to 2,000 ppm; however, it has been known to be a hazard to human health (National Academies of Science [NAS], 2010). PAA is reported to be an irritant to the upper respiratory tract, eye, and skin (Merka and Urban, 1978; Fraser and Thorbinson, 1986; Janssen, 1989; Janssen and van Doorn, 1994). Direct contact in the eye and skin can be avoided if the proper personal protective equipment (PPE) is worn, but there are limited approaches to protect the upper respiratory tract from the vapors emitted from PAA (American Thoracic Society, 1996).

Currently, there is no OSHA (Occupational Safety and Health Administration) limit on the acute or long-term exposure limit of PAA during shifts for employees. However, other governing bodies have set limits and guidelines for the exposure of PAA vapor. In 2014, the American Conference Governmental Hygienists (ACGIH) set a threshold limit of 0.04 ppm as the 15-minute Short Term Exposure Limit (STEL; American Conference of Governmental Industrial Hygienists [ACGIH], 2016). Furthermore, the National Advisory Committee for Acute Exposure Guideline Levels for Hazardous Substances (NAC/AEGLL Committee), during an 8 h exposure time, set AEGL-1, 2, and 3 limits to 0.17, 0.51, and 1.3 ppm of PAA vapor, respectively (National Academies of Science [NAS], 2010), with firm limits of total exposure time for AEGL-1 and 2 limits at 0.17, and 0.51 ppm (National Academies of Science [NAS], 2010). An exposure at AEGL-1 produces noticeable discomfort, and irritation, with reversible effects upon removal from exposure site. AEGL-2 exposure produces irreversible or other long-lasting serious health conditions and may impair one’s ability to escape. Lastly, an AEGL-3 exposure results in life-threatening health conditions and can result in death. As of 2015, NIOSH published a draft Immediately Dangerous to Life or Health (IDLH) value for 0.64 ppm (National Institute for Occupational Safety and Health [NIOSH], 2015).

As there are no current strategies employed to reduce the ambient PAA in a processing facility, there is a significant need to develop and easily implement measures to prevent PAA vapor exposure. One novel approach is to utilize the commercial aqueous ozone product (Viriditec^TM^, TetraClean Systems LLC, Omaha, NE) to distribute aqueous ozone directly to PAA, as preliminary evidence suggests, the addition of ozone can reduce the noxious nature of PAA (data not shown).

Previously, chlorine was utilized as the primary sanitizer in processing facilities but has been replaced in the last decade with PAA. Studies have demonstrated that 85 ppm of PAA has the capability to reduce the incidence of *Salmonella* and *Campylobacter* by 92 and 43% on poultry carcasses when applied in a commercial poultry chiller (Bauermeister et al., 2008b). Whereas, 30 ppm of chlorine was only capable of reducing *Salmonella* and *Campylobacter* by 43 and 13%, when used in a poultry chiller (Bauermeister et al., 2008b). Furthermore, PAA has been shown to mitigate *Staphylococcus* spp., *Listeria* spp., and generic *Escherichia coli* more than 5-log CFU regardless of the food source being evaluated (Brinez et al., 2006). Bauermeister et al. (2008a) reported the reduction of *Salmonella* and *Campylobacter* to be greater in carcasses chilled in solutions containing 200 ppm of PAA compared to those chilled in 30 ppm chlorine, ≈1 log reduction. Therefore, it is imperative to mitigate the noxious nature of PAA without reducing the bactericidal effects of PAA in poultry processing facilities. Thus, it was the objective of the current experiment to evaluate the efficacy of a commercial aqueous ozone (O_3_) alone or in combination with PAA on reducing ambient PAA and poultry pathogens when applied as an antimicrobial spray on whole chicken carcasses.

## Materials and Methods

### Viriditec^TM^ Aqueous Ozone Generation

TetraClean’s Viriditec^TM^ aqueous ozone system has been characterized as a patented technology that utilizes Nanobubble Technology to combine water and ozone to yield aqueous ozone. In the current study, the system produced ozone gas which was injected into a water stream and further infused through the systems patented configuration and mixing technology. A Q46H/64 Dissolved Ozone Monitor (Analytical Technologies Industries, Collegeville, PA, United States) was utilized to measure the specific ozone levels generated from the Viriditec^TM^ aqueous ozone system. The result was an aqueous ozone solution that contained 10 ppm of dissolved ozone in solution.

### Carcass Procurement and Indigenous Pathogen Screening

A total of 70 whole hen carcasses (7 treatments; 10 replicates) with an average weight of 1749.87 g were obtained from a free-range poultry facility immediately after processing and were void of any antimicrobial treatments prior to the onset of the current experiment. A review by the institutional animal care and use committee (IACUC) was exempted because the birds were raised in an off-campus commercial farm operation and the current study was restricted to microbiological evaluation of bird carcasses selected for study. Immediately following evisceration, on the same day all 70 carcasses were shipped on ice and upon arrival at the University of Arkansas Center for Food Safety one carcass was screened for the background indigenous presence of *Salmonella, E. coli*, and *Campylobacter*. The remaining carcasses were stored at 4°C refrigeration until the onset of the study which began within 24 h post-slaughter.

### Inocula Preparation and Inoculation

Prior to the study, a frozen stock of *Salmonella* Typhimurium (UK-1), *E. coli* (J53), and *Campylobacter jejuni* were streaked to isolation on respective media and incubated either aerobically at 37°C for 24 h or microaerophilically at 42°C for 48 h. Subsequently, one isolated colony from the incubated plates were streaked onto fresh medium and incubated under the previously mentioned conditions. Simultaneously, an isolated colony was streaked onto Xylose Lysine Deoxycholate (XLD; HiMedia, West Chester, PA, United States) and modified Charcoal-Cefoperazone-Deoxycholate agar (mCCDA; HiMedia, West Chester, PA, United States) for confirmation and incubated either aerobically at 37°C for 24 h or microaerophilically at 42°C for 48 h. Following confirmation, isolated colonies from the incubated media were then transferred to 40 mL of fresh Luria-Bertani Broth and Mueller Hinton Broth (Hardy Diagnostics, Irving, TX, United States) and incubated under previously mentioned conditions in a shaking incubator at 200 rpm for 12 to 16 h. The resulting cultures of 3 × 10^7^ CFU/mL of *Salmonella* Typhimurium (UK-1), *E. coli* (J53), and *C. jejuni*, respectively.

Directly following the overnight (12 to 16 h) incubation of the cultures, the cultures were spun down at 18,000 g for 5 min, decanted, and then washed twice in 1 × Phosphate Buffered Saline (PBS; 8 g of NaCl, 0.2 g of KCl, 1.44 g of Na_2_HPO_4_, and 0.24 g of KH_2_PO_4_ per 1 L, with the pH adjusted to 7.4 with HCl). After the final wash, the pellet was re-suspended in 400 mL of sterile PBS.

The carcasses were inoculated in a 400 mL cocktail containing 3 × 10^7^ CFU/mL of *Salmonella* Typhimurium (UK-1), *E. coli* (J53), and *C. jejuni*. *Salmonella, E. coli*, and *Campylobacter* inocula were allowed to adhere at 4°C for 60 min for a final attachment of 10^6^, 10^6^ and 10^5^ CFU/g. Following the attachment period, the whole carcass weights were recorded, and the treatments were administered. The carcasses were independently placed into a spray cabinet constructed from a modified refrigerator (Model No. FFTR1814LW2, Fridgaire, Miami, FL, United States) with four pressure nozzles that administered 500 mL of treatments via a high-pressurized spray (15 psi). The treatment was applied 4 × with 5 s on 5 s off for a total duration of 20 s treatment application. The treatments utilized in the current study were: a no-treatment negative control, tap water (TW); TW + O_3_ (10 ppm), TW + PAA (50 ppm), TW + PAA (500 ppm), TW + O_3_ + PAA (50 ppm), and TW + O3 + PAA (500 ppm). The commercial PAA utilized in the current study was Spectrum (FMC, Philadelphia, PA, United States). To reduce cross contamination, treated carcasses were placed into individual sterile poultry rinse bags (Nasco, Fort Atkinson, WI, United States) and allowed to rest for 2 min.

### Microbial Analysis

After the appropriated resting period, 400 mL of neutralizing Buffered Peptone Water (nBPW; 20.0 g of buffered peptone, 7 g of refined soy lecithin or equivalent, 1.0 g of sodium thiosulfate, 12.5 g of sodium bicarbonate, per 1 L of DI water; USDA Food Safety and Inspection Service, 2016) was poured directly on top and inside the carcasses. The carcasses were then manually agitated for 2 min in an 180° arcing motion. The carcasses were aseptically removed, discarded, and the subsequent rinsate was utilized for downstream analysis.

#### *Salmonella, E. coli*, and *Campylobacter* Enumeration

Rinsates were aliquoted to 15 mL conical tubes (VWR, Radnor, PA, United States) and subsequently 20 μL of rinsate was serially diluted to 10^-6^ in 180 μL of 1 × PBS via a flat bottom 96 well plate. The dot method was utilized in the current study where 10 μL of the rinsate was plated on XLD and mCCDA, allowed to dry completely, inverted, and incubated aerobically at 37°C for 24 h or microaerophilically at 42 °C for 48 h, respectively. On XLD, only colonies with black centers were considered as *Salmonella* and yellow colonies with surrounding yellow color change were considered as *E. coli*. On mCCDA, colony forming units with a silver metallic sheen were considered as *C. jejuni*.

### Ambient PAA

To measure the ambient PAA vapor emitted from the treatment solution application, a SafeCide ChemDAQ sensor and meter was utilized (ChemDAQ Inc., Pittsburgh, PA, United States). The sensor was located directly outside the modified spray cabinet and measurements were recorded in real-time for each treatment application (*n* = 10; *N* = 70).

### Statistical Analysis

Each carcass was randomly assigned to a treatment prior to the onset of the study. The CFU of *Salmonella, E. coli*, and *Campylobacter* were log transformed and reported on a CFU of bacteria per gram of chicken basis (CFU/g). The data were analyzed using One-Way ANOVA in JMP 14.0 (SAS Institute Inc., Cary, NC, United States). Means were separated using Tukey’s Protected HSD with a significant level of *P* ≤ 0.05.

## Results

### Quantification of *Salmonella, E. coli*, and *Campylobacter* Recovered From Treated Carcasses

**FIGURE 1:**
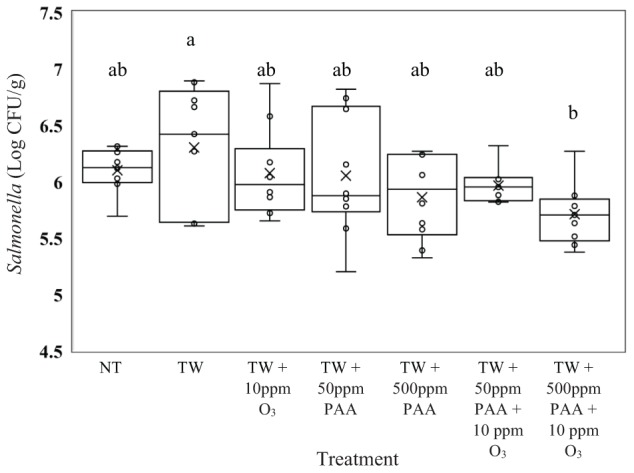
The effect of applying peracetic acid (PAA) alone and in combination with aqueous ozone at 10 ppm on the mean log CFU/g of *Salmonella* Typhimurium UK-1 on whole hen carcasses^1,2^. Carcasses were inoculated with 10^7^ CFU/mL of *Salmonella* for a final attachment of 10^6^ CFU/g of *Salmonella* Typhimurium UK-1. Birds were then placed in a modified spray cabinet to be treated for 5 s (4×) via a low pressurized spray. Immediately after, birds were rinsed in 400 mL of neutralizing buffered peptone water (nBPW) and subsequently plated to determine load of *Salmonella*. ^1^*N* = 67, *n* = 10, *P* = 0.0476. ^2^Means with different superscripts are considered significantly different (a,b).

In the current experiment, there was a treatment effect for *Salmonella, E. coli*, and *Campylobacter* recovered from the treated inoculated carcasses (*P* < 0.05). No treatments significantly reduced the concentration of *Salmonella* Typhimurium (UK-1) on whole carcasses compared to untreated carcasses (6.10 log CFU/g of *Salmonella*, Figure 1, *P* = 0.0476). However, those treated with TW + 500 ppm PAA + O_3_ (5.71 log CFU/g of *Salmonella*) had significantly lower log CFU per gram of *Salmonella* than those treated with TW alone (6.30 log CFU/g of *Salmonella*). Carcasses treated with TW + 500 ppm PAA + O_3_ (5.71 log CFU/g of *Salmonella*) did numerically possess the lowest log CFU/g of *Salmonella* compared to all other treatments. However, the treatment of both TW + PAA and TW + PAA + O_3_ did not differ significantly in recovered *Salmonella* (6.05, 5.86, 5.96, and 5.71 log CFU/g of *Salmonella*).

**FIGURE 2:**
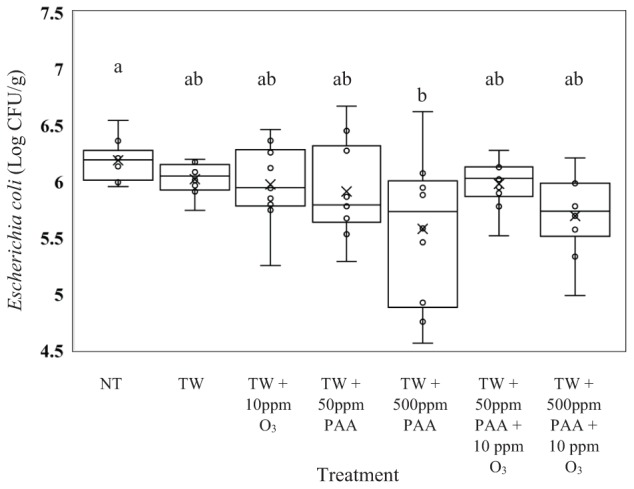
The effect of applying PAA alone and in combination with aqueous ozone at 10 ppm on the mean log CFU/g of *Escherichia coli* J53 on whole hen carcasses^1,2^. Carcasses were inoculated with 10^7^ CFU/mL of *E. coli* for a final attachment of 10^6^ CFU/g of *E. coli* J53. Birds were then placed in a modified spray cabinet to be treated for 5 s (4×) via a low pressurized spray. Immediately after, birds were rinsed in 400 mL of nBPW and subsequently plated to determine load of *E. coli*. ^1^*N* = 68, *n* = 10, *P* = 0.0126. ^2^Means with different superscripts are considered significantly different (a,b).

Unlike the recovered *Salmonella, E. coli* (J53) recovered from the rinsates of carcasses treated with TW did not exhibit significantly higher counts compared to any of the other treated carcasses (Figure 2, *P* = 0.0126). Carcasses treated with TW + 500 ppm of PAA (5.57 log CFU/g of *E. coli*) yielded a lower load of *E. coli* than those not treated (6.18 log CFU/g of *E. coli*). Similar to *Salmonella*, the recovery of *E. coli* did not differ from carcasses treated with TW + PAA and TW + PAA + O_3_, regardless of PAA concentration.

**FIGURE 3:**
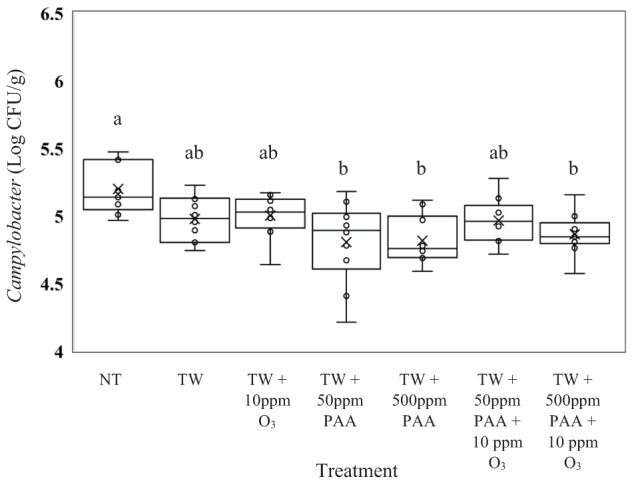
The effect of applying PAA alone and in combination with aqueous ozone at 10 ppm on the mean log CFU/g of *Campylobacter jejuni* on whole hen carcasses^1,2^. Carcasses were inoculated with 10^6^ CFU/mL of *C. jejuni* for a final attachment of 10^5^ CFU/g of *C. jejuni*. Birds were then placed in a modified spray cabinet to be treated for 5 s (4×) via a low pressurized spray. Immediately after, birds were rinsed in 400 mL of nBPW and subsequently plated to determine load of *Campylobacter*. ^1^*N* = 69, *n* = 10, *P* = 0.0006. ^2^Means with different superscripts are considered significantly different (a,b).

The recovered load of *C. jejuni* (log CFU/g of *Campylobacter*) was greatest in carcasses not treated (Figure 3; *P* = 0.0006). The *C. jejuni* recovered from carcasses not treated (5.20 log CFU/g of *C. jejuni*) did not differ from carcasses treated with TW, TW + O_3_ and TW + 50 ppm PAA + O_3_ (4.97, 5.00, and 4.96 log CFU/g of *C. jejuni*). Further, the lowest load of *C. jejuni* was recovered from carcasses treated with TW + 50 ppm PAA, TW + 500 ppm PAA, and TW + 500 ppm PAA + O_3_ (4.80, 4.81, and 4.86 log CFU/g of *C. jejuni*) which were significantly different from the untreated control.

### Quantification of PAA Vapor From Treated Carcasses

**FIGURE 4:**
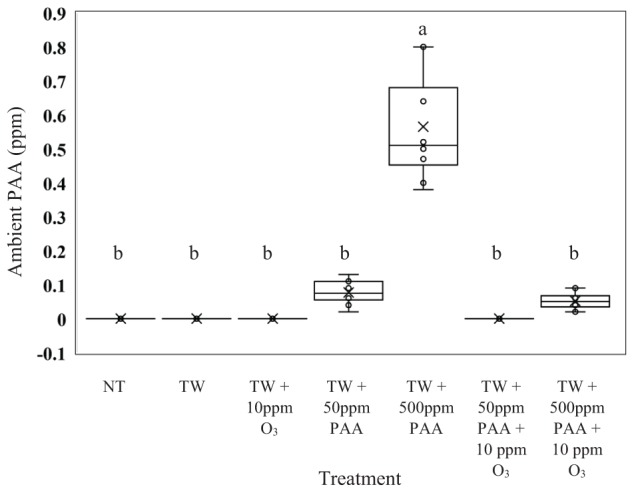
The effect of applying PAA alone and in combination with aqueous ozone at 10 ppm on whole hen carcasses on the mean ppm of the surrounding ambient PAA^1,2^. Carcasses were inoculated with *Salmonella* Typhimurium UK-1, *E. coli* J53, and *C. jejuni*. Birds were then placed in a modified spray cabinet to be treated for 5 s (4×) via a low pressurized spray. While the treatments were being applied, a ChemDaq SafeCide ambient PAA monitor and sensor, located outside the modified spray cabinet, was utilized to determine ppm of ambient PAA. ^1^*N* = 70, *n* = 10, *P* < 0.0001. ^2^Means with different superscripts are considered significantly different (a,b).

From the current experiment, it was determined that there was significant treatment effect on the production of ambient PAA (ppm) (Figure 4; *P* < 0.0001). Further, it was demonstrated that the greatest production of ambient PAA was derived from the treatment solution TW + 500 ppm PAA (0.565 ppm of Ambient PAA). The treatment solutions consisting of, NT, TW, TW + O_3_, and TW + 50 ppm PPA + O_3_ did not produce any ambient PAA; however, the ambient PAA produced from those treatments was not different that the ambient PAA produced off of the treatments: TW + 50 ppm PAA and TW + 500 ppm PAA + O_3_ (0.011 and 0.008 ppm of ambient PAA).

## Discussion

### Impact of Sanitizer Treatments on *Salmonella, E. coli*, and *Campylobacter* Inocula

In the current study, the addition of Viriditec^TM^ aqueous ozone to a commercial PAA was utilized to determine if the addition of aqueous ozone possessed synergistic affects in mitigating three Gram-negative bacteria, commonly associated with poultry. Previously, it has been suggested that Gram-negative bacteria may be more sensitive to ozone than Gram-positive bacteria due to the greater presence of peptidoglycan in the cell wall of Gram-positive bacteria. Rey et al. (1995) demonstrated the resistance to aqueous ozone was enhanced when N-acetyl glucosamine, a constituent of the peptidoglycan of bacterial cell walls, was present (pH 3 to 7).

In addition, the utilization of ozone has been demonstrated to possess the ability to disrupt the cell membrane and induce membrane permeability in *Salmonella* and *E. coli* spp., respectively, thus weakening the bacterial cell wall and ultimately leading to cell death (Komanapalli and Lau, 1996; Dave, 1999). However, in the current study, there was no difference on the reduction of any of the Gram-negative bacteria, *Salmonella, E. coli*, or *Campylobacter*, between the use of tap water or aqueous ozone (10 ppm) when sprayed for 5 s (4×) on whole hen carcasses.

The lack of effect of aqueous ozone, alone, may have been in part due to the short duration of the treatment application (5 s; 4×) utilized in the current study. Previous research has generally utilized aqueous ozone for a longer duration of exposure, when evaluating bactericidal effects of food matrices. Gertzou et al. (2016) determined the addition of gaseous ozone for 1 h at 10 ppm to fresh chicken legs extended their shelf life 4 days more than the control when packaged in polyamide/polyethylene (PA/PE) packaging. Others have seen various levels of gaseous ozone (1, 0.1, and 33 ppm) applied for 5, 20, and 9 min, respectively, to be effective at reducing *L. monocytogenes* in water, fish, and poultry samples (Fisher et al., 2000; Vaz-Vehlo et al., 2001; Muthukumar and Muthuchamy, 2013). Previously, aqueous ozone (4.5 ppm) in poultry chillers has demonstrated the potential to significantly mitigate total aerobes, psychrotrophs, coliforms, fecal coliforms, and *Salmonella* (78, 37, 91, 91, and 81%, respectively) on chicken broiler carcasses that had been chilled for 45 min compared to those not chilled (Sheldon and Brown, 1986). When aqueous ozone (0.5 to 6.5 ppm) was applied to poultry meat, in a separate study, it reduced the load of *Salmonella* Enteritidis by 0.6 to 4 log CFU (Dave, 1999).

However, ozone has demonstrated to be more effective in reducing bacteria when suspended in pure water than in food products (Khadre et al., 2001). In agreement with the current study, where aqueous ozone did not have an effect on mitigating pathogen load when utilized alone at 10 ppm, Fabrizio et al. (2002) determined that the spray application of distilled water, 10 ppm aqueous ozone, 10% trisodium phosphate (TSP), 2% acetic acid (AA), 20 ppm sodium hypochlorite, electrolyzed oxidizing water (pH 2.4 to 2.7, 1,150 mV ORP, 50 ppm free CL) on chicken whole carcasses did not have an effect on *Salmonella* Typhimurium load on day 0. However, Fabrizio et al. (2002) did report that the submersion of electrolyzed oxidizing water, TSP, AA, and aqueous ozone reduced *Salmonella* Typhimurium to levels of detection only after selective enrichment on whole chicken carcasses. The submersion of whole carcasses in aqueous ozone has also demonstrated the potential to reduce total aerobic bacteria on d 0 and reduce *E. coli* and total coliforms on d 7 compared to whole carcasses not treated (Fabrizio et al., 2002). Thus, the complete submersion of carcasses may prove to have a greater bactericidal effect than sprays may have, as observed in the current study.

In the current study when PAA was utilized alone, both concentrations of PAA, 50 and 500 ppm, reduced *C. jejuni* load, but only 500 ppm reduced *E. coli*, and no concentration of PAA reduced *Salmonella* compared to the control. Although PAA has been demonstrated to be an effective antimicrobial in previous research, mitigating pathogens by 2 logs or greater (Bauermeister et al., 2008a), the current research did not demonstrate the same efficacy. In fact, the current study demonstrated no treatments were capable of exhibiting practical reductions of pathogen load of 1 log or greater. Unlike the current research, Bauermeister et al. (2008a) demonstrated that when PAA is applied in the chiller at 200 ppm *Salmonella* and *Campylobacter* load are reduced roughly below 2 and 2.5 log CFU when artificially inoculated with 10^6^ CFU/mL of *Salmonella* and *Campylobacter*, respectively. However, as with aqueous ozone, the application method may play a part in the differences in pathogen reduction.

Other short duration antimicrobial treatments of poultry meat with PAA have demonstrated little consistency. Del Río et al. (2007) demonstrated that when chicken legs were dipped in solutions containing 220 ppm PAA (Inspexx 100, Ecolab, St. Paul, MN, United States) for 15 s, *Enterobacteriaceae* and coliforms were reduced 0.24 ± 0.19 and 0.28 ± 0.84, respectively, on d 0; however, the reduction of bacteria on legs dipped in PAA was not significantly different than the legs treated with water. In contrast, Nagel et al. (2013)found that the post-chill application of PAA at concentration 400 and 1,000 ppm for 20 s had the potential to reduce the load of *Salmonella* Typhimurium and *C. jejuni* on artificially contaminated chicken breasts by 2 log CFU/mL.

Although, aqueous ozone and PAA, alone, did not mitigate pathogens as previous studies have shown, the combination of aqueous and PAA demonstrated an additive effect. This additive effect may be in part due to the byproducts of PAA and O_3_. As PAA is the equilibrium product of acetic acid and hydrogen peroxide, when it dissociates acetic acid and hydrogen peroxide molecules are released. Hydrogen peroxide in aqueous solution is then capable of partially dissociating to hydroperoxide anion (HO_2_^-^) which is very reactive to ozone (Taube and Bray, 1940). Further, as acetic acid directly affects the pH, ozone is stabilized as ozone is more stable at a low pH (Khadre et al., 2001).

Overall, the current study demonstrated that the reduction of pathogens while utilizing the modified spray cabinet, was significant, it was not extensive. Previously, it has been demonstrated that bacteria reside not only on the exposed muscle surfaces, but within the feather follicles (Barnes and Impey, 1968). Thus, creating difficulties for antimicrobial treatments to properly disinfect poultry carcasses. This may explain the small reductions seen in the current study. This was also seen in a study performed by Sheldon and Brown (1986), who demonstrated less than a 1 log reduction of total aerobic bacteria, psychrotrophs, coliforms and fecal coliforms, and *Salmonella* when broiler carcasses were chilled in aqueous ozone for 45 min compared to those not chilled.

### Decomposition of PAA Vapor

In the current study, the addition of aqueous ozone to PAA reduced the ambient PAA emitted when carcasses were treated in a modified spray cabinet. There is limited research on the proposed mechanism behind the reduction of PAA vapor, however, the authors have two proposed hypotheses to describe reduction in ambient PAA vapor. First, PAA (CH_3_COOOH) is formed from the equilibrium of hydrogen peroxide (H_2_O_2_ or OH) and acetic acid (CH_3_COOH). From the reaction of the acetic acid radical (Reaction 1) and ozone (O_3_, Reaction 2) result in the formation of the peroxyacetic acid radical which disproportionates (Reaction 3) to produce 70% hydrogen peroxide (H_2_O_2_) and other products: formaldehyde, glyoxylic acid, glycolic acid, and organic peroxides (Sehested et al., 1992) as seen in the following reactions:

$$\mathrm{OH}+{\mathrm{CH}}_{3}\mathrm{COOH}\to^{\prime }{\mathrm{CH}}_{2}\mathrm{COOH}+H_{2}O$$

$$\prime {\mathrm{CH}}_{2}\mathrm{COOH}+O_{3}\to^{\prime }{\mathrm{OOCH}}_{2}\mathrm{COOH}$$

$$2^{\prime }{\mathrm{OOCH}}_{2}\mathrm{COOH}\to 70\%\;H_{2}O_{2}+\mathrm{other}\;\mathrm{products}$$

The second explanation for the loss of ambient PAA vapor is that the ozone is being “robbed” an oxygen from the PAA to reduce it to acetic acid in the gas state, preventing the OH radical formation as seen in the following reaction:

$${\mathrm{CH}}_{3}\mathrm{OOOH}+O_{3}\to {\mathrm{CH}}_{3}\mathrm{OOH}+2O_{2}$$

## Conclusion

In conclusion, the combination of 10 ppm of aqueous ozone, Viriditec^TM^, and 500 ppm of PAA has the potential to mitigate the presence of *Salmonella* Typhimurium (UK-1), *E. coli* J53, and *C. jejuni*. Furthermore, the combination of 10 ppm of aqueous ozone with 500 ppm of PAA demonstrated the ability of ozone to reduce the ambient PAA vapor by 90%, when compared to 500 ppm of PAA alone. Thus, the application of TetraClean’s product Viriditec^TM^ has the ability to enhance the safety for poultry processing employees. Although the current study demonstrated the promising capabilities of aqueous ozone and PAA, in combination, future research is necessary to develop an understanding of the impact the combination of aqueous ozone and PAA has on the shelf life of processed poultry and the subsequent changes in the microbiome.

## Author Contributions

All authors significantly contributed to the work of the current study. KF, DD, and SR designed and prepared the current study with the assistance from DW, BP, and MD. DD and DW conducted the experiments. DD analyzed the data and constructed the manuscript with the assistance from KF, DW, BP, MD, and SR.

## Conflict of Interest Statement

BP, DW, and MD are employed by the company TetraClean Systems LLC, Omaha, NE, United States. The remaining authors declare that the research was conducted in the absence of any commercial or financial relationships that could be construed as a potential conflict of interest.
